# Teleost fish IgM^+^ plasma-like cells possess IgM-secreting, phagocytic, and antigen-presenting capacities

**DOI:** 10.3389/fimmu.2022.1016974

**Published:** 2022-09-26

**Authors:** Liting Wu, Yanjian Yang, Along Gao, Jun Li, Jianmin Ye

**Affiliations:** ^1^ Guangdong Provincial Key Laboratory for Healthy and Safe Aquaculture, Institute of Modern Aquaculture Science and Engineering, School of Life Sciences, South China Normal University, Guangzhou, China; ^2^ Guangdong Provincial Engineering Technology Research Center for Environmentally-Friendly Aquaculture, Guangzhou Key Laboratory of Subtropical Biodiversity and Biomonitoring, School of Life Sciences, South China Normal University, Guangzhou, China; ^3^ Laboratory for Marine Fisheries Science and Food Production Processes, Laboratory for Marine Biology and Biotechnology, Pilot National Laboratory for Marine Science and Technology, Qingdao, China; ^4^ School of Science and Medicine, Lake Superior State University, Sault Sainte Marie, MI, United States

**Keywords:** teleost fish, anterior kidney, IgM^+^ B cell, antibody-secreting, phagocytosis, antigen-presentation

## Abstract

Plasma cells are terminally differentiated antibody-secreting B lymphocytes that contribute to humoral immunity by producing large numbers of antibodies. Increasing evidence suggests that teleost fish B cells share certain characteristics with mammalian B1 B cells, including antibody-secreting, phagocytic, and antigen-presenting capacities. However, the difference between mature B cells and plasma cells remains unclear. In this study, we found that, based on their light-scattering characteristics, tilapia anterior kidney (AK) leukocytes can be categorized into two IgM^+^ B-cell subsets: the lymphoid (L) gate and granulocyte–monocyte/macrophage (G-M) subsets. G-M gate cells are more numerous than L-gate cells and have higher mean fluorescence, but lower forward scatter and side scatter. We analyzed the morphological and ultrastructural features of sorted IgM^+^ cells and found that L-gate IgM^+^ cells have a high nucleus–cytoplasm ratio and lymphocyte-like morphology, whereas G-M gate IgM^+^ cells have a small nucleus, more abundant endoplasmic reticulum, and a larger number of mitochondria, and have a plasma cell-like or macrophage-like morphology. To further characterize the cell types, we examined the specific patterns of expression of B-cell- and T-cell-related genes. We found that B-cell-specific genes were expressed by both L-gate and G-M gate IgM^+^ cells, and that G-M gate IgM^+^ cells secreted extremely high levels of IgM. However, T-cell-related genes were highly expressed only in L-gate IgM^–^ cells. These results suggest that G-M gate IgM^+^ cells are similar to plasma-like cells, with high antibody-secreting capacity. Given that G-M gate cells include the granulocyte, monocyte, and macrophage cell types, but not B cells, monocyte/macrophage markers were used to investigate the cell types further. A macrophage receptor with a collagenous structure was frequently observed, and macrophage-expressed gene-1 was highly expressed, in the G-M gate IgM^+^ cells. Phagocytic capacity, as determined by ingestion of beads or bacteria, was significantly higher in G-M gate IgM^+^ cells than in L-gate IgM^+^ cells, as was antigen-processing capacity. Our findings show that tilapia AK leukocytes can be divided into two IgM^+^ B-cell subsets and that G-M gate IgM^+^ cells resemble plasma-like cells, having high antibody-secreting, phagocytic, and antigen-presenting capacities. Thus, this study increases our understanding of the functions of teleost fish plasma-like cells.

## Introduction

Teleost fish, like mammals, have an adaptive immune system (comprising cell-mediated immunity and humoral immunity) to protect them against pathogen infection (1). By producing antibodies, the humoral immune system effectively and constantly monitors the areas of the fish that are susceptible to pathogenic invasion (2). B lymphocytes are at the center of humoral immunity, secreting specific antibodies against invasive pathogens. The developmental pathways of teleost fish B cells remain largely unknown; however, using highly conserved transcription factors, B-cell subsets are characterized as pro-B, pre-B, or immature/mature B cells, plasmablasts, or plasma cells (3–7). Plasmablasts and plasma cells are classified as antibody-secreting cells (ASCs) and synthesize and secrete immunoglobulins (Igs) (5). IgM is the most common immunoglobulin produced by teleosts, and its heavy chain and mechanism of action are similar to those of mammalian IgM (8, 9). However, teleost B cells also produce two other immunoglobulins with different heavy-chain isotypes, known as IgD and IgT (10–13). All three immunoglobulins occur in both membrane and secreted forms, and play a role in both systemic and mucosal immunity (12–15). Lymphoid tissues in teleosts include the spleen and posterior kidney, which are secondary lymphoid organs containing abundant mature B cells (5, 7, 16). However, the highest concentration of developmental forms of B cells conditioning specific humoral immunity are found in the teleost anterior kidney (AK). Furthermore, as a functional counterpart of mammalian bone marrow, where B cells proliferate, develop, and mature (2, 5, 7), the AK is considered to be the major lymphoid organ in teleosts and the primary site for fish hemopoiesis. As such it has it has been used extensively to explore B-cell development and activation (5–7, 17). In zebrafish, mononuclear phagocytes (monocytes/macrophages and dendritic cells) and hematopoietic cells from whole kidney can be characterized by flow cytometry, according to their light-scattering characteristics and forward and side light scatter (FSC/SSC) parameters, as lymphoid or myeloid cells (4, 18–20). In other teleost fish, AK leukocytes can be divided by flow cytometry into a smaller lymphocyte-like [lymphoid (L) gate] population and a larger myeloid population [granulocyte–macrophage (G-M) gate] population (21, 22). It has been reported that IgM^+^ B lymphocytes can be considered L-gate rather than G-M gate cells on the basis of their FSC and SSC characteristics (21).

The discovery of the phagocytic and microbicidal abilities of teleost fish B cells has caused researchers to explore the relationship between mammalian B lymphocytes and macrophages (14). In mammals, B cells are classified as conventional, B-2, cells and B1 cells, which can be further divided into B1-a (CD5^+^) and B1-b (CD5^–^) subtypes. B1-a cells play a role in innate immunity by producing natural antibodies, whereas B1-b cells are critical in the development of IgM memory cells (23, 24). Mouse B-2 cells originate mainly from bone marrow, whereas B1 cells originate mainly from fetal liver and to a lesser extent from adult bone marrow (25). Murine B1 cells have active phagocytic capabilities and act as a bridge between innate and adaptive immunity (26, 27). The identification of trout CD5 provides additional evidence for the similarities between fish IgM^+^ B cells and mammalian B1 cells (28). There is now increasing evidence that fish B cells share some phenotypical and functional characteristics of mammalian B1 B cells, such as phagocytic and antigen-presenting capacity (27–32). Furthermore, trout IgM^+^ cells can respond to CD40L (a thymus-dependent activation signal), which indicates that teleost IgM^+^ cells have similar functions to mammalian B2 cells (33). Owing to the limited availability of antibody reagents for fish immune molecules, different B-cell subtypes cannot be identified and sorted further. However, plasma cells can be identified by their phenotype, as they are larger and have a lower membrane IgM level than other types of B cells (4, 34–36). These phenotypic characteristics develop when naive B cells are activated and differentiate into plasmablast/plasma cells (33, 37, 38). In addition, surface expression of major histocompatibility complex (MHC) class II is reduced when unstimulated B cells differentiate into IgM ASCs, indicating a decrease in the antigen-presenting capacity of B cells (39).

Our recent study of a single-cell transcriptome of AK leukocytes from Nile tilapia (*Oreochromis niloticus*) found that AK leukocytes comprise myeloid cells and lymphocytes, and that B cells can divided into subpopulations based on their differentiation stage (40). Among these B-cell subpopulations are terminally differentiated plasma cells, which secrete a large quantity of antibodies. During our exploration of B-cell heterogeneity in tilapia AK, we discovered the existence of a population of IgM^+^ G-M gate cells. Theoretically, IgM^+^ B lymphocytes are L-gate cells, whereas G-M gate cells consist of monocytes, macrophages, and granulocytes. Recently, a study on carp IgM^+^ cells revealed the existence of an IgM^+^ myeloid cell subset of plasma-like cells (22). This phenomenon has also been reported in other studies of teleost fish IgM^+^ B cells, but the existence of G-M gate IgM^+^ cells has not been analyzed further (21, 41). Given the similarities between B cells and macrophages, in particular the fact that both cell types are capable of phagocytosis, it was thought that B cells might have evolved from ancient phagocytic cells (14). Therefore, we hypothesize that G-M gate IgM^+^ cells have different functions from L-gate IgM^+^ B cells.

In this context, by comparing cell size and patterns of expression of B-cell/macrophage-related genes in L-gate and G-M gate IgM^+^ B cells, we established that G-M gate IgM^+^ B cells resemble plasma-like B cells and have the capacity to secrete large amounts of IgM antibodies. Furthermore, we investigated the phagocytic capability of both L-gate and G-M gate IgM^+^ cells, and established that G-M gate IgM^+^ cells have higher phagocytic capacity than L-gate IgM^+^ B cells. Finally, we confirmed that G-M gate IgM^+^ cells have greater antigen-presenting capacity than L-gate IgM^+^ B cells. These results provide new insights and show that teleost plasma cells, as well as having high antibody-secreting ability, have phagocytic and antigen-presenting capacities.

## Materials and methods

### Experimental fish

Nile tilapia (*Oreochromis niloticus*), with a mean body weight of 450 g (± 50 g), were supplied by Guangdong Tilapia Breeding Farm (Guangzhou, China) and maintained at the aquaculture breeding center of the South China Normal University (35). Before experiments, the Nile tilapia were maintained in 300-L fiberglass-reinforced plastic tanks in an automatic filtering aquaculture system for 3 weeks at 28°C (± 2°C), with a 12 h/12 h light/dark photoperiod. All animal protocols were reviewed and approved by the University Animal Care and Use Committee of the South China Normal University (SCNU-SLS-2021-009).

### Anterior kidney leukocyte isolation

Nile tilapia were anesthetized with 3-aminobenzoic acid ethyl ester (MS-222; Aladdin), and blood was extracted from the caudal vein into a heparinized syringe to prevent blood pollution of the AK. Leukocytes from the AK were obtained using previously published methods, with some modification (42). In summary, the AK was dissected using aseptic dissection tools and placed in a sterile plastic culture dish containing 5  ml of Roswell Park Memorial Institute (RPMI)-1640 medium (Gibco, Shanghai, China) supplemented with 100 units/ml penicillin and 100 μg/ml streptomycin (P/S; Life Technologies, Carlsbad, California, USA). The tissue was repeatedly aspirated using a 1- ml syringe until no large pieces of tissue remained. Next, the single-cell suspension was filtered through a 100-μm nylon cell strainer (BD Biosciences) to remove the tissue fragments. The cell suspension was made up to a volume of 10  ml with RPMI-1640 medium, and was then layered on the same volume of Histopaque 1077 (Sigma-Aldrich, Darmstadt, Germany) in a 50 -ml centrifuge tube, before being centrifuged at 500 × g for 40 minutes at 4°C. Leukocytes were collected from the interface layer and washed three times with RPMI-1640 medium. Cell quantity and viability were determined by 0.4% trypan blue (Sigma-Aldrich, Darmstadt, Germany), and cells were resuspended to a concentration of 1 × 10^7^ cells/ml in RPMI-1640 medium containing 10% fetal bovine serum (FBS) (Gibco, Shanghai, China).

### Flow cytometry and cell sorting

**Figure 1:**
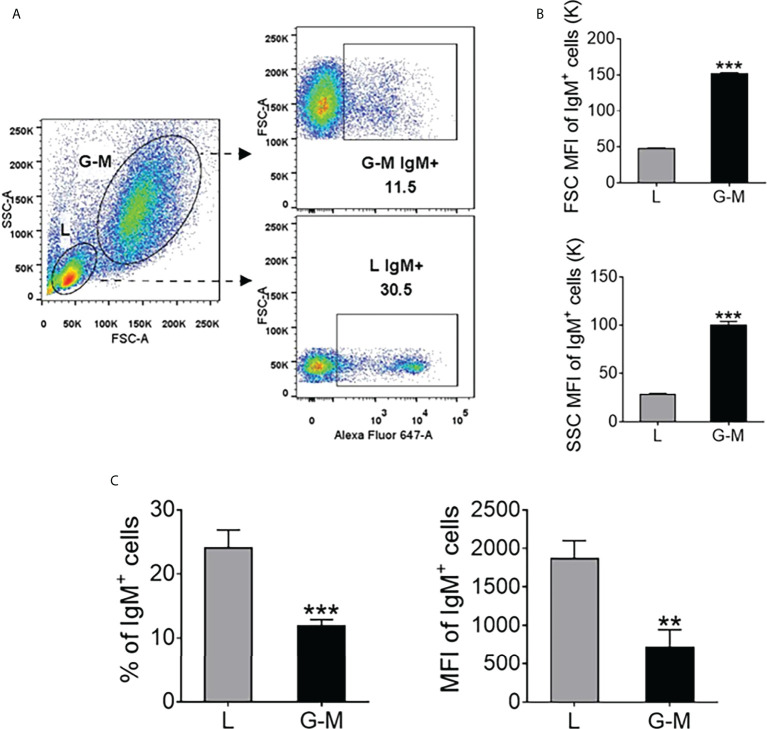
The distribution of IgM^+^ cells in Nile tilapia anterior kidney **(AK)** leukocytes determined by flow cytometry. **(A)** Leukocytes were extracted from the AK with Ficoll-Paque^®^ PREMIUM (density 1.077 g/ml) and were stained with Alexa Fluor™ **(AF)** 647-labeled mouse anti-IgM monoclonal antibody (mAb); the leukocytes were then classified into L-gate and G-M gate cells. The percentage of IgM^+^ cells in these two gates is shown in the scatter profiles. **(B)** Forward scatter (FSC) and sideward scatter (SSC) mean fluorescence intensity (MFI) value of IgM^+^ cells in L and G-M gates. The MFI for each parameter was quantified and is shown on bar plots as mean value ± standard error of mean (SEM) (*n* = 6 fish). **(C)** The percentage and MFI of IgM^+^ cells in L and G-M gates. Asterisks indicate significant differences (**p ≤ 0.01 and ***p ≤ 0.001).

AK leukocytes from Nile tilapia were incubated for 30 minutes at 4°C with an anti-tilapia IgM monoclonal antibody (mAb) (0.25 µg/ml), which was labeled with Alexa Fluor™ (AF) 647 Thermo Fisher Scientific, Waltham, Massachusetts, USA, in RPMI-1640 containing 5% FBS (35). After washing three times with RPMI-1640 medium, cells were resuspended and analyzed by fluorescence-activated cell sorting (FACS) using a BD FACSAria™ III flow cytometer (BD Biosciences). Both IgM^+^ and IgM^–^ cells from different gates, i.e., the L and G-M gates, were analyzed based on their forward scatter (FSC) and sideward scatter (SSC) profiles using FlowJo V10 software (TreeStar), Tree Star, USA, as shown in **Figure 1**. IgM^+^ and IgM^–^ cells (5 × 10^6^ cells/ml) were sorted and collected from both L and G-M gates. The purity of the various sorted cell populations was analyzed, as shown in **Figure S1**. The sorted cells showed a higher purity level (> 95%). They were collected in TRIzol reagent (Vazyme Biotech, Nanjing, China) and were immediately frozen using liquid nitrogen, before being stored at –80°C for further total RNA isolation.

### Confocal microscopy, Giemsa staining, and transmission electron microscopy of L-gate and G-M gate IgM^+^ cells

IgM^+^ cells from the L and G-M gates were studied using confocal microscopy to compare cell size and fluorescence intensity. In summary, IgM^+^ cells (1 × 10^6^) were sorted from L and G-M gates, as described above. The cells were then stained with Hoechst 33342 solution (Thermo Fisher Scientific, Waltham, Massachusetts, USA) at a concentration of 20 µM at room temperature (RT) and were protected from light for 10 min. The cells were washed with RPMI-1640 medium and seeded on poly-L-lysine-coated slides for 30 min. Laser-scanning confocal microscopy images were acquired with a Zeiss LSM 710 confocal microscope (Carl Zeiss, Jena, Germany). For Giemsa staining, the IgM^+^ cells obtained from the L and G-M gates were dyed with Giemsa stain solution (Solarbio Science, Beijing, China) in accordance with the manufacturer’s instructions, then observed and images captured with a Leica DM6 camera (Leica Microsystems, GmbH, Germany). The sorted cells were fixed in 2.5% glutaraldehyde solution and 1.0% osmium tetroxide. Then, after gradual dehydration in acetone, the samples were embedded in epoxy resin in accordance with standard procedures. Ultrathin sections were prepared after 72 hours of polymerization at 80°C and stained with uranyl acetate and lead citrate for subsequent examination. Cell images were observed and recorded using a FEI Tecnai transmission electron microscope (FEI Company, Hillsboro, OR, USA).

### Real-time PCR analysis of flow cytometry-isolated leukocyte populations

Total RNA from sorted IgM^+^ and IgM^–^ cells, as described above, was extracted using the TRIzol reagent kit (Vazyme) in accordance with the manufacturer’s instructions, and RNA pellets were eluted from the columns in RNase-free water (35). The quality and quantity of the extracted total RNA were determined by the NanoDrop™ 2000 assay (Thermo Fisher Scientific). This extracted total RNA was used for cDNAs synthesis using reverse transcription cDNA synthesis kits (Yeasen, Shanghai, China). The cDNAs were diluted 10-fold and stored at –80°C for real-time PCR analysis. To evaluate the heterogeneity of IgM^+^ and IgM^–^ cells from L and G-M gates, the transcriptional levels of B-cell-, T-cell-, and G-M-related genes were detected with specific primers (see **Table S1**). This was carried out using the SYBR™ Green dye method in a volume of 20 µl, which contained 10 µl of 2 × SYBR mix (Yeasen) (Shanghai, China), 2 µl of forward primer, and 2 µl of reverse primer (0.1 μm), 3 µl of diluted cDNA, and 3 µl of double-distilled water using the CFX96 Touch Real-Time PCR System (Bio-Rad Laboratories, Hercules, CA, USA). Each sample was analyzed in duplicate under the following conditions: 3 min at 95°C, followed by 40 amplification cycles (15 s at 95°C and 1 min at 60°C). A melting curve for each PCR analysis was determined by reading fluorescence at every degree between 60°C and 95°C to ensure that only a single product had been amplified. The expression of individual genes was normalized to the relative expression of tilapia β-actin and the expression levels were calculated using the 2^–Δ^ *^Ct^* method, where Δ*Ct* was determined by subtracting the β-actin value (**Table S1**) from the target *Ct*, as previously described (35).

### IgM antibody-secreting capacities of L- and G-M-gate IgM^+^ cells

L-gate and G-M gate IgM^+^ cells from Nile tilapia, AK were sorted as described above. The collected IgM^+^ cells (1 × 10^6^) were plated in 96-well flat-bottom plates (Nunc, Waltham, Massachusetts, USA) in 200 μl of RPMI-1640 medium, which was supplemented with 10% FBS and P/S, before being cultured at 25°C for 3 days. The plate was centrifuged at 500 × g for 5 min at 4°C, and the supernatant was collected and stored at –80°C until we measured the amount of IgM using western blotting. The samples were electrophoresed on 12% sodium dodecyl sulfate-polyacrylamide gel electrophoresis (SDS-PAGE) gels under reducing conditions and were transferred to 0.22-μm polyvinylidene fluoride membranes (PVDF) (Millipore, Darmstadt, Germany). The membranes were washed three times with Tris-buffered saline (25 mM Tris-HCl, 150 mM NaCl, pH 7.5) supplemented with 0.1% Tween 20 (TTBS; Sigma), followed by blocking with 0.5% bovine albumin (BSA) (Sigma-Aldrich) in TTBS for 1 h at 37°C. After being washed three times, the membranes were covered with mouse anti-IgM mAb (0.5 µg/ml) at RT for 1 h (35). After being washed three times, secondary antibodies labeled with horseradish peroxidase (HRP), goat anti-mouse IgG mAb (1 : 2000; Southern Biotech, Birmingham, Alabama, USA), were added to cover the membrane for 1 h at RT. After being washed three times, the protein was visualized by Tanon 5200 Multi (Ewell Biotechnology, Shanghai, China) with the BeyoECL Plus (Beyotime, Shanghai, China). Image J software (National Institutes of Health, Bethesda, MD, USA) was used to analyze the data.

### Phagocytosis assays

The phagocytic activity of tilapia IgM^+^ cells was measured as reported in our previous study (35). Briefly, AK leukocytes were isolated as described above, the concentration of cells was adjusted to 1 × 10^6^ cells/100 μl in a 1.5-ml tube, and cells were incubated with 0.5-μm- or 1.0-μm-diameter Fluoresbrite^®^ Yellow Green Microspheres (YG beads; Polysciences Inc., Warrington, PA, USA) in a 1 : 10 (cell–bead) ratio. The numbers of *Streptococcus agalactiae* (*S. agalactiae*), *Aeromonas hydrophila* (*A. hydrophila*), and *Escherichia coli* (*E. coli*) were counted, and the bacteria were then heat inactivated at 60°C for 40 min, labeled with fluorescein isothiocyanate (FITC) (Sigma-Aldrich) and subjected to flow cytometry, as described in our previous report (35). Cells were incubated with FITC-conjugated bacteria in a 1 : 50 (cell–bacterium) ratio. The mixture of cells was incubated at 25°C for 3 hours. Then cells were then collected, and the non-ingested beads or bacteria were removed by centrifuging twice at 100 × g for 10 min at 4°C. After washing with RPMI-1640 medium, leukocytes were stained with mouse anti-tilapia IgM mAb (0.25 μg/ml) labeled with AF647 for 30 min at 4°C. After washing twice at 500 × g for 10 min at 4°C, 0.4% trypan blue (Sigma-Aldrich) was added to the cell suspension to quench fluorescence from the remaining cell surface-bound bacteria, and the cells were then analyzed with a BD FACSAria III flow cytometer. Cells in both L and G-M gates were analyzed. The analysis was also carried out with FlowJo V10 software.

### Antigen-processing assay

The antigen-processing capacity of IgM^+^ B cells was measured using the EnzChek Protease Assay Kit (Invitrogen, Carlsbad, California, USA), as reported in previous studies (33, 43, 44). To summarize, AK leukocytes from tilapia at a concentration of 2 × 10^6^ cells/ml, obtained as described above, were incubated with green fluorescent BODIPY DQ-Casein™ at 5 μg/ml for 1 h. The cells were then washed three times with FACS staining buffer (Miltenyi Biotec; GmbH, Germany) and incubated with AF647-anti-IgM mAb (0.25 μg/ml) for 30 min at 4°C (35). After being washed three times, the cells were analyzed by flow cytometry as described above.

### Statistical analysis

Data handling, statistical analyses and graphic representation were carried out using GraphPad Prism version 8.02 (GraphPad Software Inc., San Diego, CA, USA). Statistical analyses were performed to compare values obtained in each experimental group using the two-tailed Student’s *t*-test with Welch’s correction when the *F*-test indicated that the variances of both groups differed significantly. Differences between the mean values were considered significant at different levels (*p ≤ 0.05, **p ≤ 0.01, and ***p ≤ 0.001).

## Results

### IgM^+^ cells from L and G-M gates have different phenotypic characteristics

Based on their light-scattering characteristics, cells obtained from zebrafish whole kidney marrow, including erythroid cells, lymphoid cells, precursor cells, myeloid cells, and eosinophils, were gated (19, 45). Zebrafish leukocytes can be gated as lymphoid cells, precursor cells, and granulocytes (4), as well as lymphoid cells and myeloid cells/progenitors (18). In rainbow trout, based on the known cell types in each gate, kidney leukocytes have been gated as L-gate and G-M gate cells (21). In this study we analyzed the AK leukocytes from Nile tilapia. As shown in **Figure 1**, both L-gate and G-M gate IgM^+^ cells were identified, in different proportions, the proportion L-gate cells (24.04% ± 2.83%) being significantly higher than the proportion of G-M gate cells (11.92% ± 0.97%) (**Figure 1C**, left), as was the mean fluorescence intensity (MFI) of the IgM^+^ cells (**Figure 1C**, right). Furthermore, the MFI, in terms of both FSC and SSC, was significantly higher in G-M gate IgM^+^ cells than in L-gate IgM^+^ cells (**Figure 1B**). When mature B cells differentiate into plasmablast/plasma cells, the membrane Ig levels decrease but Ig secretion increases, and an increase is observed in the overall size of the cells, as reflected by FSC on flow cytometry (5, 37, 46, 47). The characteristics reported above suggest that the IgM^+^ cells in the L gate are similar to naive B cells, and the IgM^+^ cells in the G-M gate share more similarities with plasmablasts/plasma cells.

### Comparison of cell morphology of L-gate and G-M gate IgM^+^ cells

**Figure 2:**
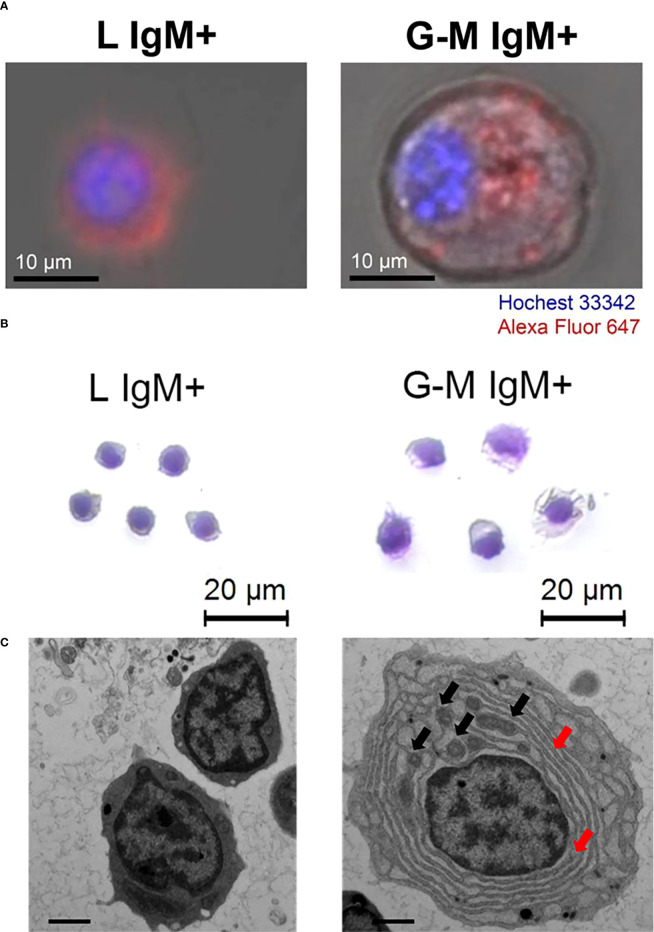
Morphological characterization and ultrastructural features of tilapia IgM^+^ cells from L and G-M gates. L-gate and G-M gate IgM^+^ cells were sorted from anterior kidney (AK) leukocytes and stained with 647-labeled mouse anti-IgM mAb using flow cytometry, as shown in **Figure S1**, and were visualized and observed with **(A)** a Zeiss LSM 710 confocal microscope, **(B)** hematoxylin and eosin staining, and **(C)** transmission electron microscopy. For ultrastructural analysis, the representative cells were magnified 6000-fold and the scale bar represents 1 μm. Black arrowheads indicate mitochondria and red arrowheads indicate endoplasmic reticulum.

To visualize the IgM^+^ cells from L and G-M gates, we separated the cells using flow cytometry. The IgM^+^ cells were then observed and analyzed by laser-scanning confocal microscopy, as well as by Giemsa staining (**Figure 2**). The purity of the isolated populations after sorting was more than 95% (see **Figure S1**). We captured the bright field and fluorescence field and overlapped these two images . The IgM^+^ cells from the L gate were characterized as being smaller and having brighter fluorescence than G-M gate cells (**Figure 2A**), which was consistent with the flow cytometry results. Furthermore, we analyzed the sorted IgM^+^ cells with Giemsa staining, which showed that L-gate IgM^+^ cells had lymphocyte-like morphology, whereas G-M gate IgM^+^ cells had monocyte/macrophage or plasma-like morphology (**Figure 2B**). The ultrastructure of the sorted cells showed that G-M gate IgM^+^ cells had a lower nucleocytoplasmic ratio, a more abundant endoplasmic reticulum, and relatively more mitochondria than L-gate IgM^+^ cells (**Figure 2C**).

### Expression patterns of B-cell-, T-cell-, and monocyte/macrophage-related genes on L-gate and G-M gate leukocytes

**Figure 3:**
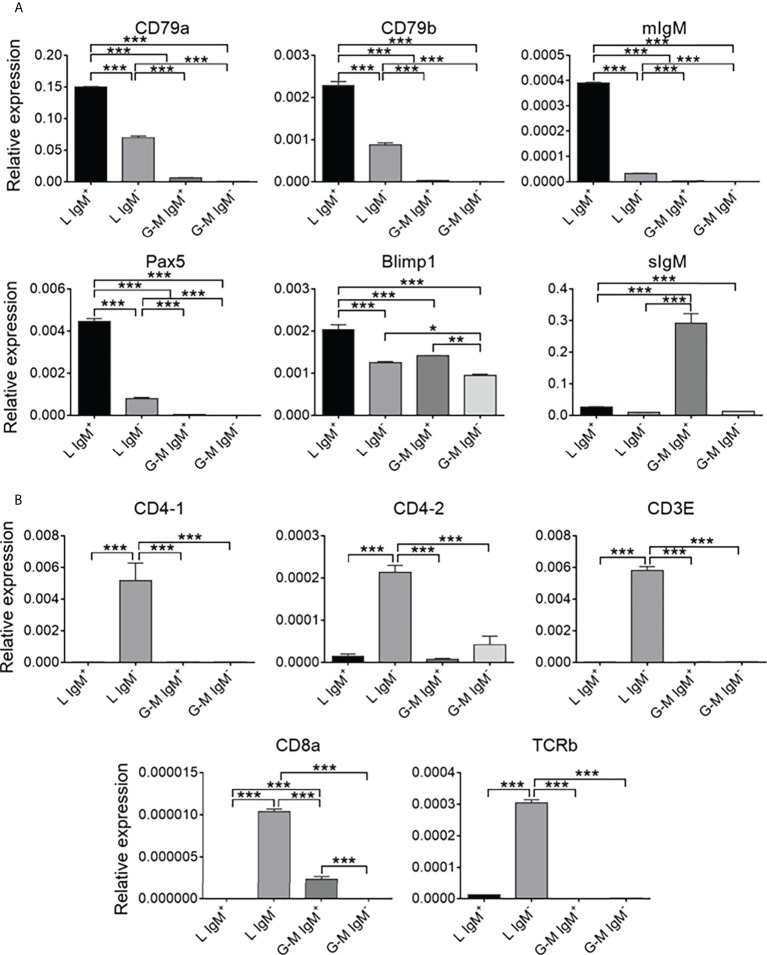
Comparison of the expression of B-cell- and T-cell-related genes among tilapia L-gate IgM^+^, L-gate IgM^–^, G-M gate IgM^+^, and G-M gate IgM^–^ cells from anterior kidney (AK) leukocytes. **(A)** The expression patterns of B cell-related genes: *CD79a*, *CD79b*, membrane IgM (*mIgM*), paired box protein 5 (*PAX5*), B-lymphocyte-induced maturation protein 1 (*BLIMP1*), and secreted IgM (*sIgM*). **(B)** The expression patterns of T-cell-related genes: *CD4-1*, *CD4-2*, *CD3E*, *CD8a*, and *TCRb* (*n* = 5 fish). Asterisks indicated significant differences, where *p ≤ 0.05, **p ≤ 0.01, and ***p ≤ 0.001.

**Figure 4:**
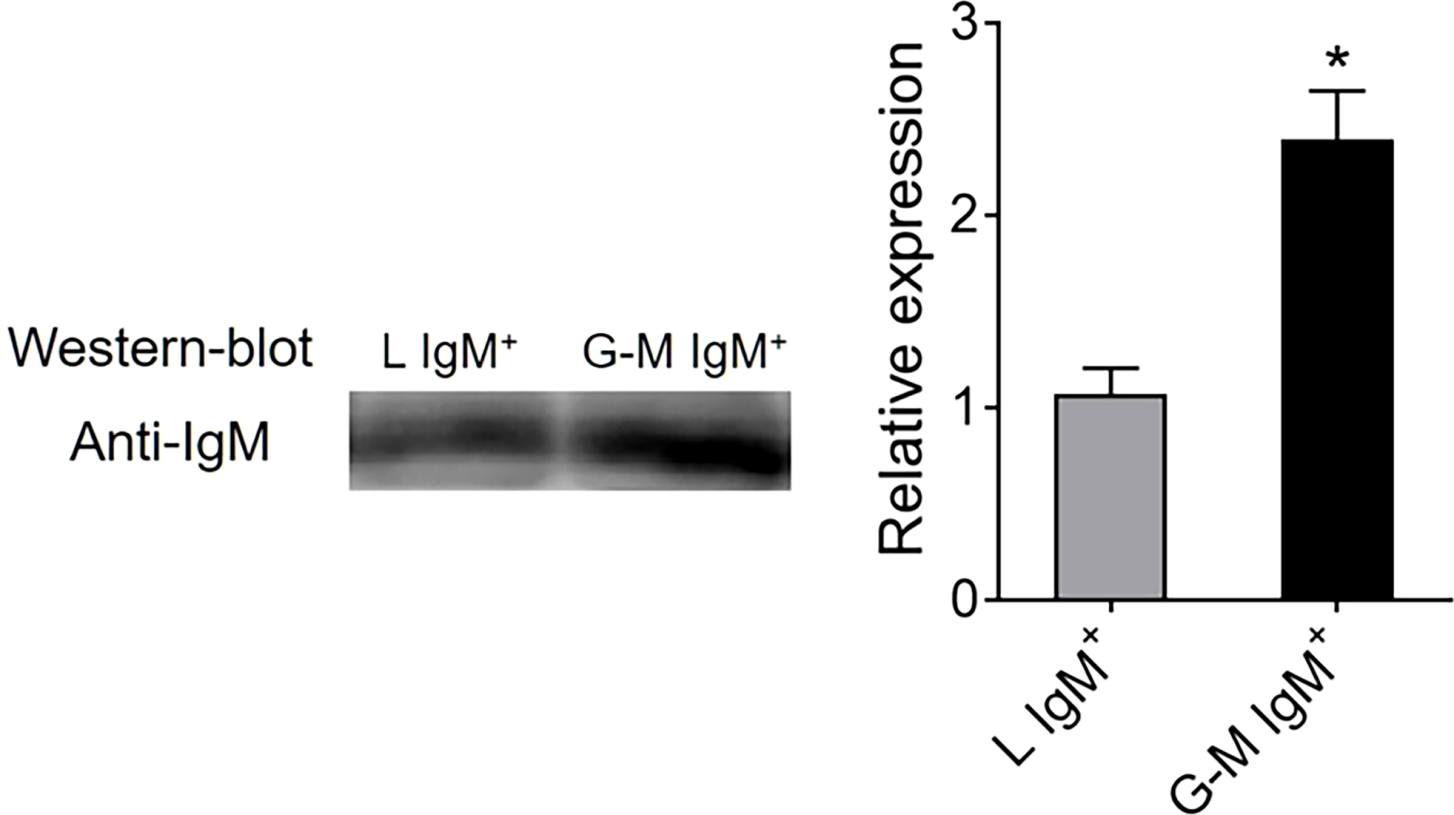
Detection of IgM secretory capacity between tilapia L-gate IgM^+^ and G-M gate IgM^+^ cells. L-gate IgM^+^ and G-M gate IgM^+^ cells were sorted from tilapia anterior kidney (AK) leukocytes using a BD FACSAria™ III flow cytometer, before being plated in flat-bottom 96-well plates in 200 μl of Roswell Park Memorial Institute (RPMI)-1640 medium supplemented with 10% fetal bovine serum (FBS) and 100 units/ml penicillin and 100 μg/ml streptomycin (P/S). After being cultured *in vitro* for 72 hours at 25°C, the supernatants were collected to measure the amount of secreted IgM using western blotting (*n* = 3 fish). *p ≤ 0.05.

**Figure 5:**
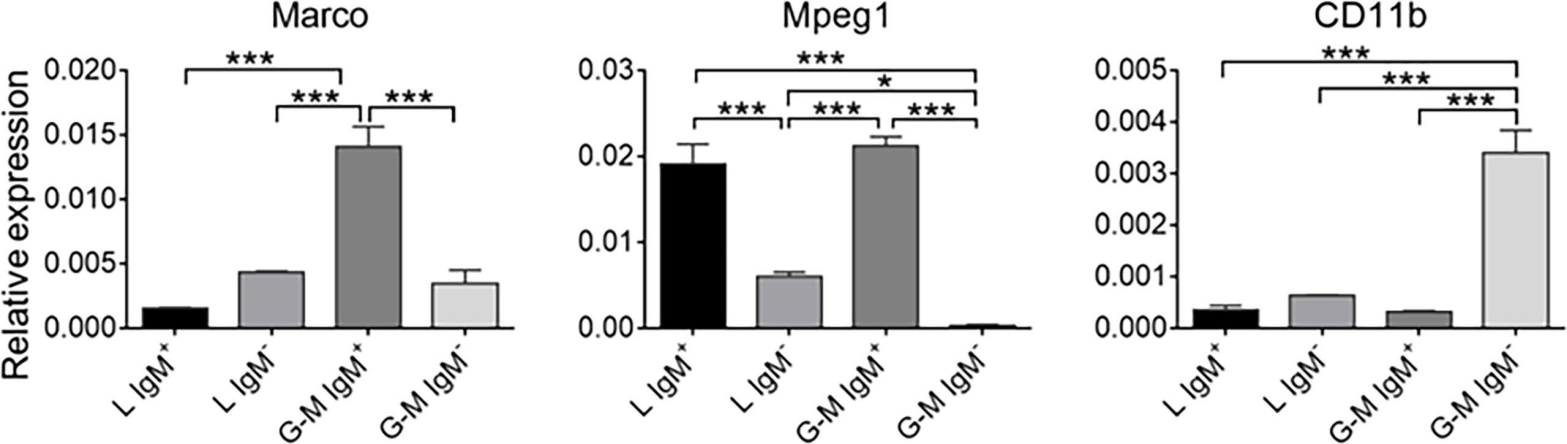
Expression patterns of granulocyte- and macrophage-related genes—*MARCO*, *MPEG1*, and *CD11b*—in tilapia L-gate IgM^+^, L-gate IgM^-^, G-M gate IgM^+^, and G-M gate IgM^–^ cells (*n* = 6 fish). Asterisks indicate significant differences (*p ≤ 0.05 and ***p ≤ 0.001).

To further identify the cell types from L and G-M gates, we first analyzed the transcription levels of B-cell- and T-cell-related genes. As shown in **Figure 3A**, B-cell-related genes, including *CD79a*, *CD79b*, membrane IgM (*mIgM*), paired box 5 (*PAX5*), B-lymphocyte-induced maturation protein 1 (*BLIMP1*), and secreted IgM (*sIgM*), were highly expressed in both L-gate and G-M gate IgM^+^ cells. In particular, *sIgM* was found to be highly expressed in G-M gate IgM^+^ cells, indicating that these cells have significant antibody-secreting capacity (**Figure 4**) and suggesting that G-M gate IgM^+^ cells tend to be plasmablasts or plasma-like cells. T-cell-related genes were expressed only in L-gate IgM^–^ cells, with *CD4-1* and *CD3E* being the genes most commonly expressed (**Figure 3B**). Given that G-M gate cells comprise granulocytes, monocytes, and macrophages, we further analyzed and compared the granulocyte and monocyte/macrophage marker genes, including the macrophage receptor with collagenous structure (*MARCO*), macrophage-expressed gene 1 (*MPEG1*), and *CD11b* genes. Expression of *MARCO* and *MPEG1* was higher in G-M gate IgM^+^ cells, whereas expression of *CD11b* was greater in G-M IgM^–^ cells (**Figure 5**).

### Phagocytosis of L-gate and G-M gate IgM^+^ B cells

**Figure 6:**
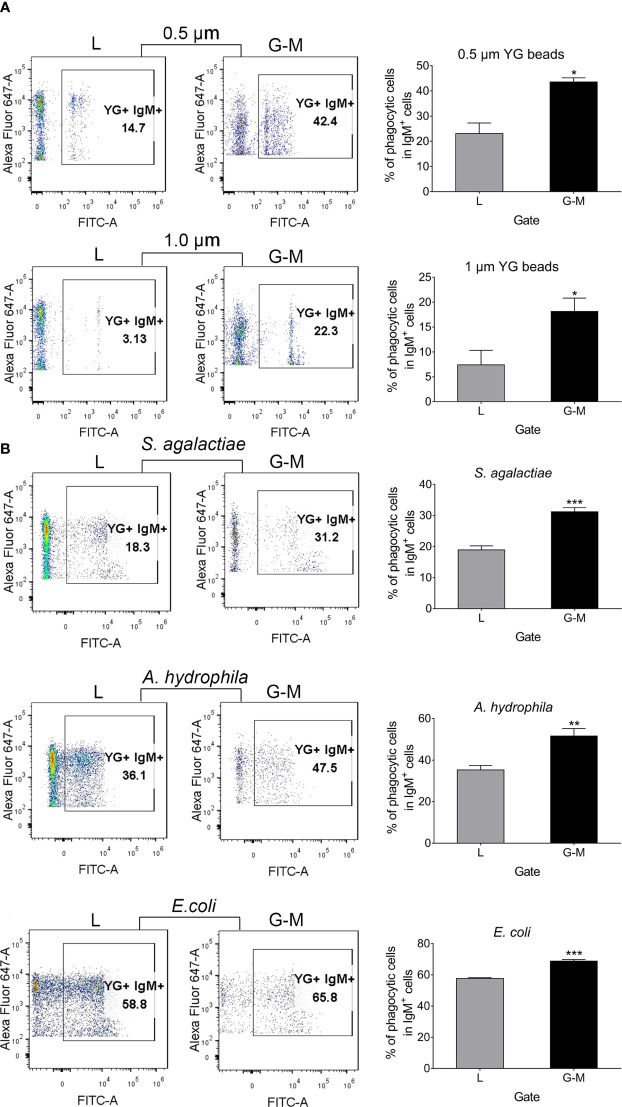
Tilapia L-gate IgM^+^ and G-M gate IgM^+^ cell phagocytosis of fluorescent beads (0.5 µm and 1.0 µm in diameter) and different pathogens (including *S. agalactiae*, *A. hydrophila*, and *E. coli*. **(A)** For the phagocytosis of beads, leukocytes from anterior kidney (AK) were incubated with 0.5-µm- and 1.0-µm-diameter fluorescent beads in a 1 : 10 (cell–bead) ratio. **(B)** For the pathogens, leukocytes from AK were incubated with inactivated *S. agalactiae*, *A. hydrophila*, and *E. coli* labeled with fluorescein isothiocyanate (FITC); the cell-to-pathogen ratio was 1 : 100. After 3 h, cells were dyed with Alexa Fluor™ (AF) 647-labeled mouse anti-IgM mAb for 30 min, and were then washed three times with Roswell Park Memorial Institute (RPMI)-1640 medium at 4°C for 5 min. Cells were detected with a BD FACSAria™ III flow cytometer and analyzed with FlowJo V10 software. Scatterplots and the statistical results (phagocytic IgM^+^ cells as a percentage of total IgM^+^ cells in each gate) are shown (*n* = 6 fish). Asterisks indicate significant differences (*p ≤ 0.05, **p ≤ 0.01, and ***p ≤ 0.001.

Teleost fish B cells have potent *in vitro* and *in vivo* phagocytic activities, which supports the idea that B cells evolve from an ancestral phagocytic cell type (14). Our previous study indicated that cell differentiation affects B-cell phagocytic activities, decreasing the phagocytic capacity but not the phagocytic ability of peripheral blood IgM^+^ cells (35). Given our finding that cell markers specific for macrophages (*MARCO* and *MPEG1*) were highly expressed in G-M gate IgM^+^ cells, it was not surprising to find that both L-gate and G-M gate IgM^+^ cells exhibit phagocytic capacity. AK leukocytes were incubated with 0.5-μm- or 1.0-μm-diameter YG fluorescent beads for 3 h in a 1 : 10 (cell–bead) ratio, and were then labeled with AF647-labeled anti-IgM mAb; the IgM^+^ cells from the L gate and G-M gate were sorted and analyzed. In the presence of both 0.50-μm- and 1.0-μm-diameter fluorescent beads, G-M gate IgM^+^ cells showed significantly greater phagocytic capacity than L-gate IgM^+^ B cells (**Figure 6A**). To explore their capacity to ingest bacteria, AK leukocytes were incubated with two pathogenic bacteria (*S. agalactiae* and *A. hydrophila*) isolated from Nile tilapia and a non-pathogenic bacterium (*E. coli*). Similar results were observed in that the G-M gate IgM^+^ cells phagocytosed a significantly higher percentage of bacteria than did the L-gate IgM^+^ cells (**Figure 6B**).

### Antigen-presenting capacity of L-gate and G-M gate IgM^+^ cells

**Figure 7:**
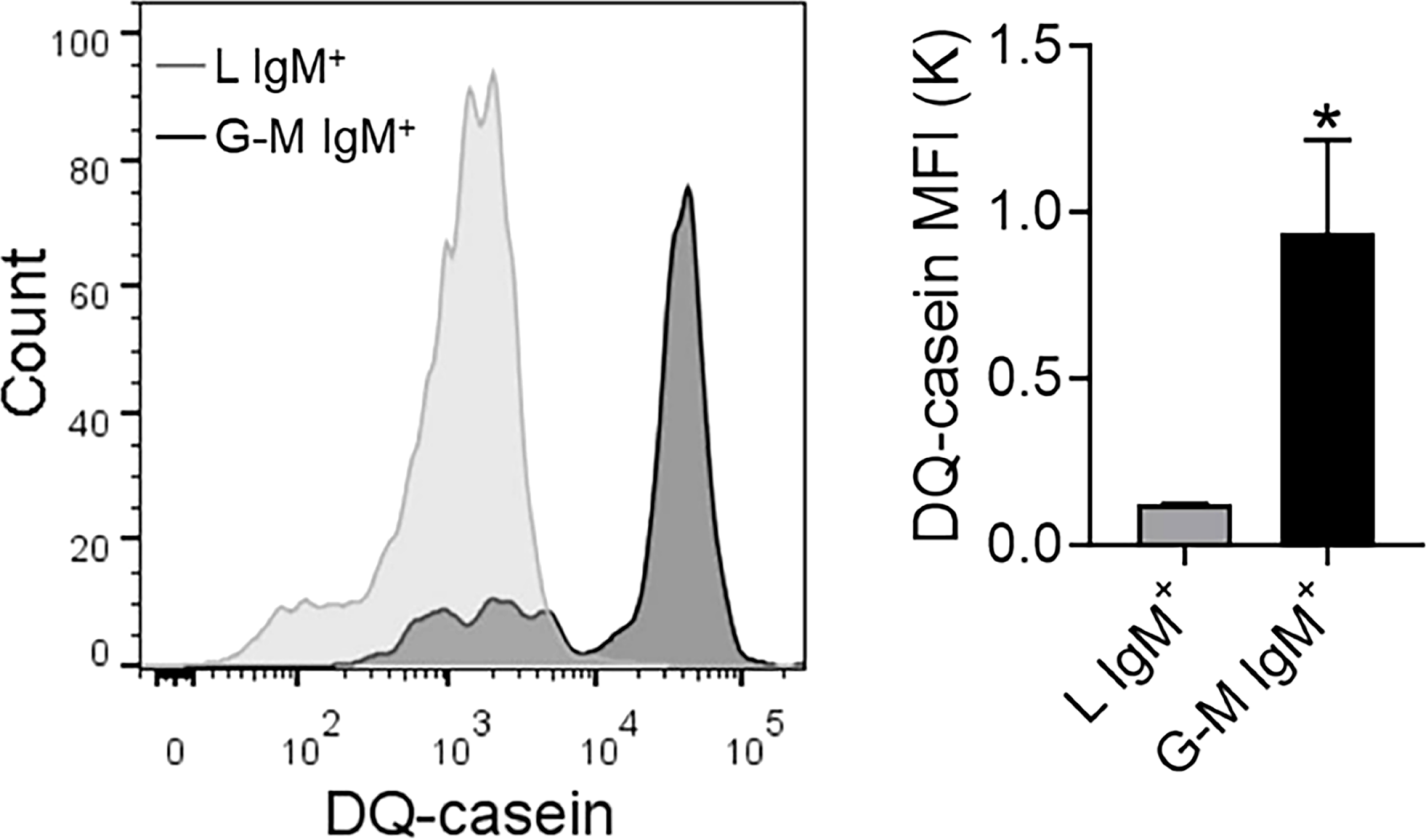
Antigen-processing capacity of L-gate and G-M gate IgM^+^ cells. Nile tilapia anterior kidney (AK) leukocytes were incubated with DQ-casein (5 μg/ml) for 1 h at room temperature (RT) then labeled with an anti-tilapia IgM mAb and analyzed by flow cytometry. IgM^+^ cells in the L and G-M gates were sorted and the signal intensity was quantified. Representative histograms are shown on the left panel, and the DQ-casein mean fluorescence intensity (MFI) from different individual fish (*n* = 3 fish) is shown in the right panel. Asterisks indicate significant differences (*p ≤ 0.05).

Teleost fish IgM^+^ cells, being antigen-presenting cells, play a pivotal role in the initiation of the adaptive immune response (32). Considering the high phagocytic capacity of G-M gate IgM^+^ cells, we examined the antigen-presenting capacity of both types of IgM^+^ cell using EnzChek^®^ Protease Assay Kits (Invitrogen). In this assay, which has been employed to investigate the protease-mediated antigen-processing capacities of fish, antigen-processing capacity is positively correlated with fluorescence (43, 44, 48). When cells were labeled with DQ-casein, it was observed that the MFI for DQ-casein was significantly higher in G-M gate IgM^+^ cells than in L-gate IgM^+^ cells (**Figure 7**), demonstrating that G-M gate IgM^+^ cells have greater antigen-presenting capacity than L-gate IgM^+^ cells.

## Discussion

A large amount of evidence has shown that there are phenotypic and functional similarities between fish IgM^+^ B cells and mammalian B1 cells, especially after the discovery of B-cell phagocytosis (14, 28, 30, 33). In mammalian species, murine B1 B cells from the peritoneal and pleural cavities can differentiate into mononuclear phagocyte-like cells *in vitro*, and perform phagocytosis *in vivo* (26, 49), suggesting that B1 B cells may evolve from phagocytic predecessors that are involved in the innate phase of immune responses in higher vertebrates. A developmental relationship has been established between B1 cells and macrophages, with the identification of biphenotypic B-cell/macrophage progenitors in the fetal liver and adult bone marrow (50, 51). Furthermore, splenic B1-a (CD5^+^) cells become phagocytes when co-cultivated with fibroblasts, also named B/macrophages (52). Continuous research has increased our knowledge of mammalian B1 B-cell distribution, and it has been established that B1 B cells exist in different tissues (53). Recently, research has demonstrated that mature IgM-expressing plasma cells resident in the bone marrow express a membrane functional B-cell receptor that regulates the signaling pathway and senses antigens and product cytokines on antigenic challenge (54, 55). Given that teleost fish do not have bone marrow or lymph nodes, their AK plays an essential role in hematopoiesis stem cell development and differentiation. Being the major organ for B-cell maturation and IgM production, the AK has a vital role in systemic immunity. Plasma cells are the terminally differentiated mature B lymphocytes that secrete large numbers of protective antibodies against a broad spectrum of pathogens. In a previous study, we identified that B-cell subpopulations exist in the AK; however, we did not further analyze B-cell heterogeneity among (40). In the current study, we identified an IgM^+^ plasma-like cell that not only secretes a large number of antibodies, but also phagocytoses foreign bodies and pathogens and presents antigens, which provides a better understanding of the resemblance between teleost IgM^+^ B cells and mammalian B1 cells.

It is known that mature B cells exhibit a high nucleus–cytoplasm ratio, little rough endoplasmic reticulum (RER), and an uncondensed nucleus. In the process of differentiating into a plasmablast, a mature B cell undergoes a dramatic increase in size and experiences a decrease in or even total loss of surface IgD/IgM. Plasma cells have a small, dense, eccentric nucleus, voluminous cytoplasm containing a prominent amount of RER, and an enlarged Golgi body to facilitate continuous antibody secretion (56). Plasma cells are larger size and of greater internal complexity than naive/mature B cells, which show greater FSC and SSC on flow cytometry. Furthermore, teleost fish plasma cells have higher IgM-secreting capacity, lower membrane IgM, higher levels of *BLIMP1* transcription, and lower levels of *PAX5* transcription than naive/mature B cells (6). Our results demonstrate that tilapia AK G-M gate IgM^+^ cells tend to be plasma-like cells with high antibody-secreting capacity, whereas L-gate IgM^+^ cells resemble mature/naive B cells. The high levels of transcription of T-cell-related genes in L-gate IgM^–^ cells indicate that L-gate IgM^–^ cells are T cells, as reported previously (40). Similarly, it has been previously demonstrated in zebrafish or rainbow trout that L-gate cells are lymphocytes (B cell and T cell), whereas G-M gate cells consist of granulocytes and monocytes (4, 18, 20, 21). However, in grass carp, IgM^+^ plasma cells are a subset of myeloid cells, being larger and having a smaller nucleus–cytoplasm ratio and more endoplasmic reticulum and mitochondria than lymphoid IgM^+^ cells (22). Our results are consistent with these findings in grass carp; however, there are differences, such as higher IgM^+^ MFI in G-M gate IgM^+^ cells than in L-gate IgM^+^ cells, that are consistent with the mRNA expression pattern of *mIgM*. In rainbow trout, G-M gate IgM^+^ cells were shown in the data of the flow chart, despite not being discussed (21). Therefore, we assume that the plasma-like G-M gate IgM^+^ cell is a universal phenomenon in teleost fish. The number of studies on teleost fish B has been increasing; however, there is limited focus on plasmablast and plasma cells. Our research provides a better understanding of teleost fish plasma cells and suggests that this subject warrants further research.

The finding of teleost fish IgM^+^ cell phagocytosis supports the study of mammalian peritoneal cavity B1-cell phagocytic and intracellular killing abilities (27, 31). It has been suggested that B cells evolve from macrophages or ancient phagocytic cells. Our previous study on the phagocytic capabilities of L-gate IgM^+^ B cells from tilapia demonstrated that B-cell differentiation decreases phagocytic capacity, but does not affect phagocytic ability (35). The finding is not contradicted by the discovery, reported here, that G-M gate IgM^+^ plasma-like cells have significant phagocytic capability. We have also analyzed peripheral blood and found, based on IgM fluorescence intensity, that, as in peritoneal cavity, L-gate cells are naive B cells and plasma cells (34). In this study, the IgM^+^ plasma-like cells that were identified were in the G-M gate, and exhibited significant differences from L-gate cells. In addition, mammalian B1 cells are capable of phagocytosis, microbicidal activity, and IgM secretion. Murine B1 cells maintain the characteristics of both lymphoid and myeloid lineages, and can even differentiate into phagocytes (23, 31). Furthermore, bone marrow CD5^–^ plasma cells can express surface IgM and produce natural IgM (57, 58) and IgM^+^ plasma cells sense antigens and retain roles in antigen presentation and pathogen clearance (54, 59). Given that teleost fish IgM^+^ B cells resemble mammalian B1 B cells, it is, therefore, understandable that fish G-M gate IgM^+^ plasma-like cells in the AK have high phagocytic and antigen-presenting capacities. Whether or not the antibody produced by G-M gate IgM^+^ cells is natural IgM, and the difference, if any, between IgM antibodies secreted by L-gate IgM^+^ cells and G-M gate IgM^+^ cells, need to be further investigated. In addition, whether or not CD40L stimulates L-gate IgM^+^ cells and G-M gate IgM^+^ cells should be further investigated, as this would provide a better understanding of the relationship between teleost fish B cells and mammalian B2 cells.

In conclusion, we discovered and identified tilapia IgM^+^ plasma-like cells in the AK with IgM-secreting, phagocytic, and antigen-presenting capacities. This discovery provides us with better understanding of the resemblance of teleost IgM^+^ B cells to mammalian B1 cells. The findings described in this study may have important implications for the perception of teleost fish B cells in humoral immunity as well as in innate immune response.

## Data availability statement

The original contributions presented in the study are included in the article/**Supplementary Material**. Further inquiries can be directed to the corresponding author.

## Ethics statement

All animal protocols were reviewed and approved by the University Animal Care and Use Committee of the South China Normal University (SCNU-SLS-2021-009).

## Author contributions

LW performed and analyzed all the experiments with the help from AG and YY. AG provided support in cell sorting. YY inactivated the bacterium and labeled them with fluorescein. JY and LW designed the experiments. JL contributed to suggestion and critical reading of the manuscript. LW wrote the main body of the manuscript, and JY revised and edited the final manuscript. All the authors contributed to the article and approved the submitted manuscript.

## Funding

This study was supported by the National Natural Science Foundation of China (32102827 and 31972818), the Natural Science Foundation of Guangdong Province, China (2019A1515012065), China Postdoctoral Science Foundation (2019M662959), and Guangdong Basic and Applied Basic Research Foundation (2019A1515110987). The author gratefully acknowledges the support of 2022 International (Regional) Cooperation and Exchange Programs of SCNU.

## Conflict of interest

The authors declare that the research was conducted in the absence of any commercial or financial relationships that could be construed as a potential conflict of interest.

## Publisher’s note

All claims expressed in this article are solely those of the authors and do not necessarily represent those of their affiliated organizations, or those of the publisher, the editors and the reviewers. Any product that may be evaluated in this article, or claim that may be made by its manufacturer, is not guaranteed or endorsed by the publisher.
