# Supplementary material for: Teleost fish IgM+ plasma-like cells possess IgM-secreting, phagocytic, and antigen-presenting capacities
Source: Front Immunol. 2022 Sep 26;13:1016974. doi: 10.3389/fimmu.2022.1016974 (PMC9550268; doi:10.3389/fimmu.2022.1016974)
Supplement: Supplementary file 1 [file Presentation_1.pptx]

## Slide 1
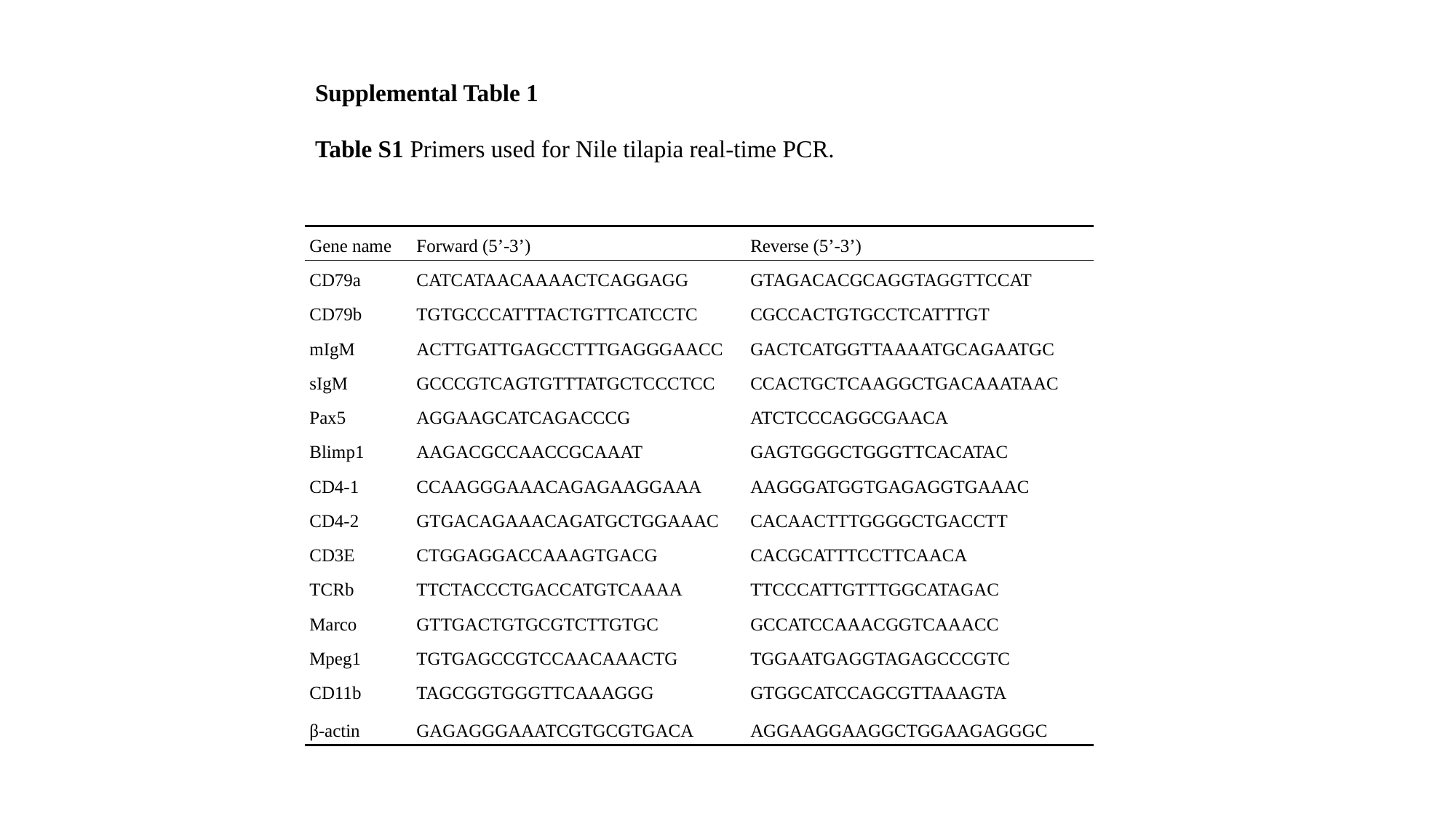

Supplemental Table 1
Table S1 Primers used for Nile tilapia real-time PCR.
| Gene name | Forward (5’-3’) | Reverse (5’-3’) |
| --- | --- | --- |
| CD79a | CATCATAACAAAACTCAGGAGG | GTAGACACGCAGGTAGGTTCCAT |
| CD79b | TGTGCCCATTTACTGTTCATCCTC | CGCCACTGTGCCTCATTTGT |
| mIgM | ACTTGATTGAGCCTTTGAGGGAACC | GACTCATGGTTAAAATGCAGAATGC |
| sIgM | GCCCGTCAGTGTTTATGCTCCCTCC | CCACTGCTCAAGGCTGACAAATAAC |
| Pax5 | AGGAAGCATCAGACCCG | ATCTCCCAGGCGAACA |
| Blimp1 | AAGACGCCAACCGCAAAT | GAGTGGGCTGGGTTCACATAC |
| CD4-1 | CCAAGGGAAACAGAGAAGGAAA | AAGGGATGGTGAGAGGTGAAAC |
| CD4-2 | GTGACAGAAACAGATGCTGGAAAC | CACAACTTTGGGGCTGACCTT |
| CD3E | CTGGAGGACCAAAGTGACG | CACGCATTTCCTTCAACA |
| TCRb | TTCTACCCTGACCATGTCAAAA | TTCCCATTGTTTGGCATAGAC |
| Marco | GTTGACTGTGCGTCTTGTGC | GCCATCCAAACGGTCAAACC |
| Mpeg1 | TGTGAGCCGTCCAACAAACTG | TGGAATGAGGTAGAGCCCGTC |
| CD11b | TAGCGGTGGGTTCAAAGGG | GTGGCATCCAGCGTTAAAGTA |
| β-actin | GAGAGGGAAATCGTGCGTGACA | AGGAAGGAAGGCTGGAAGAGGGC |

## Slide 2
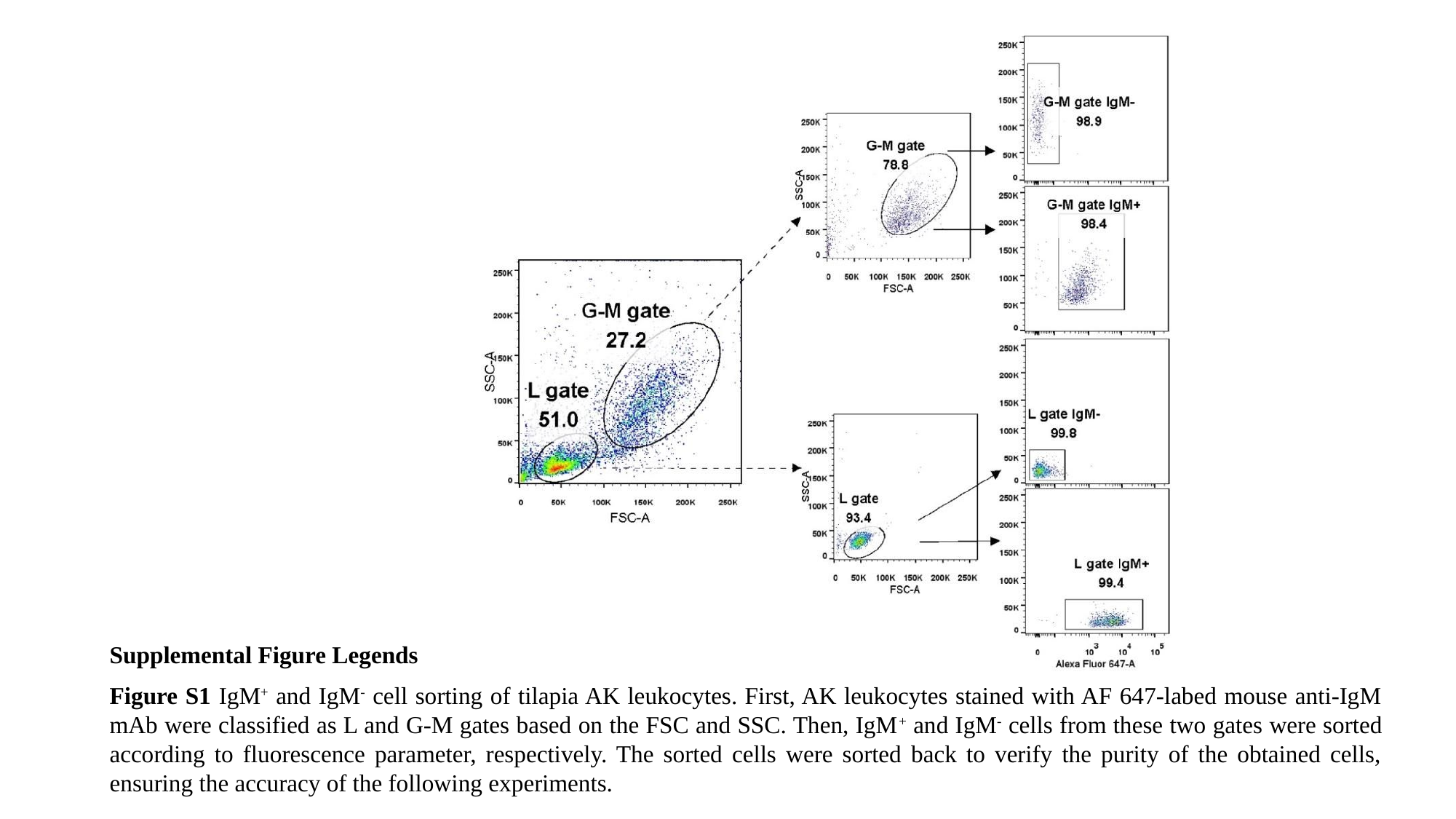

Supplemental Figure Legends
Figure S1 IgM+ and IgM- cell sorting of tilapia AK leukocytes. First, AK leukocytes stained with AF 647-labed mouse anti-IgM mAb were classified as L and G-M gates based on the FSC and SSC. Then, IgM+ and IgM- cells from these two gates were sorted according to fluorescence parameter, respectively. The sorted cells were sorted back to verify the purity of the obtained cells, ensuring the accuracy of the following experiments.
